# [Retracted] miRNA-30d serves a critical function in colorectal cancer initiation, progression and invasion via directly targeting the GNA13 gene

**DOI:** 10.3892/etm.2025.13013

**Published:** 2025-11-10

**Authors:** Shan Muhammad, Qingchao Tang, Liu Wei, Qian Zhang, Guiyu Wang, Bilal Umar Muhammad, Kavanjit Kaur, Tatiana Kamchedalova, Zhao Gang, Zheng Jiang, Zheng Liu, Xishan Wang

Exp Ther Med 17:260–272, 2019; DOI: 10.3892/etm.2018.6902

Following the publication of this paper, it was drawn to the Editor’s attention by a concerned reader that, for the migration and invasion assay experiments shown in Fig. 4, the ‘SW480/Mimic’ (migration assay) panel in Fig. 4A appeared to show an area of overlap with the ‘SW480/Mimic’ invasion panel in Fig. 4C; in addition, overlapping data panels were also identified in the Transwell migration and invasion assay data shown in Fig. 6C. Upon performing an independent analysis of the data in this paper in the Editorial Office, it was subsequently revealed that some of these same data, in addition to the tumor images shown in Fig.. 7B, had already been submitted for publication in articles written by different authors at different research institutes.

In view of the fact that the abovementioned data had already apparently been published previously, the Editor of *Experimental and Therapeutic Medicine* has decided that this paper should be retracted from the Journal. The authors were asked for an explanation to account for these concerns, but the Editorial Office did not receive a reply. The Editor apologizes to the readership for any inconvenience caused.

